# A case report of lymphoplasmacytic lymphoma with spherocytosis

**DOI:** 10.1515/biol-2025-1286

**Published:** 2026-03-02

**Authors:** Tianyu Ma, Tingting Liu, Bolin Yuan, Liang Wang, Guoqiang Liu

**Affiliations:** School of Clinical Medicine, Shandong Second Medical University, Weifang, China; Shengli Oilfield Central Hospital, Shandong, China

**Keywords:** lymphoplasmacytic lymphoma, spherocytosis, complications, treatment, case report

## Abstract

This article presents a case of a rare lymphoplasmacytic lymphoma (LPL) complicated by spherocytosis in a 74-year-old male. The patient reported progressive fatigue and anemia and had a medical history of type 2 diabetes, hypertension, and cerebral infarction. Laboratory tests indicated moderate anemia (hemoglobin 80 g/L) and a monoclonal increase in serum IgG. A bone marrow biopsy combined with immunohistochemistry confirmed the diagnosis of lymphoplasmacytic lymphoma (IgG-κ type, MYD88 L265P negative). A peripheral blood smear revealed an increase in spherocytes, a positive acidified glycerolysis test (AGLT50), abnormal erythrocyte osmotic fragility, and a negative direct antiglobulin test. Genetic screening for hereditary erythrocyte diseases showed no pathogenic variations. The patient’s condition stabilized following targeted therapy with zanubrutinib and rituximab (ZR regimen). This case underscores the complexity of diagnosing dual hematological anomalies, highlights the importance of multidisciplinary collaboration, and seeks to explore the potential pathophysiological link between LPL and spherocytosis, offering a reference for diagnosis and treatment in similar clinical scenarios.

## 1 Introduction

Lymphoplasmacytic lymphoma (LPL) is a rare form of low-grade malignant B-cell lymphoma, marked by the clonal proliferation of lymphocytes and plasma cells in tissues like bone marrow and lymph nodes. Most LPL cases are also classified as Waldenstrom macroglobulinemia (WM), characterized by the secretion of monoclonal ne immunoglobulin (Ig) M. However, some LPL cases do not involve IgM, and certain patients lack the MYD88 L265P mutation, indicating significant heterogeneity in LPL’s molecular mechanisms and clinical presentations [1]. Spherocytosis is another hematological disorder, traditionally caused by hereditary spherocytosis (due to mutations in genes like ANK1 and SPTB) or acquired autoimmune hemolytic anemia (mediated by anti-red blood cell antibodies) [2], 3]. When a patient presents with both spherocytosis and B-cell lymphoma, yet tests negative on the direct antihuman globulin test (Coombs test) and lacks a clear genetic background, determining the cause becomes challenging. In such cases, spherocyte formation might be linked to the indirect effects of malignant tumors, such as disrupting the bone marrow microenvironment and inducing chronic inflammation, though this connection remains unclear. This article discusses a rare case of IgG-κ type, MYD88 L265P-negative LPL combined with spherocytosis. The case is unique because the patient exhibits no typical hemolytic symptoms. By examining the diagnosis and treatment of this case, we aim to highlight the complexity of diagnosing dual hematological abnormalities and explore the potential non-antibody-dependent pathogenic mechanisms linking LPL and acquired red blood cell membrane defects.

## 2 Case report

**Figure 1:**
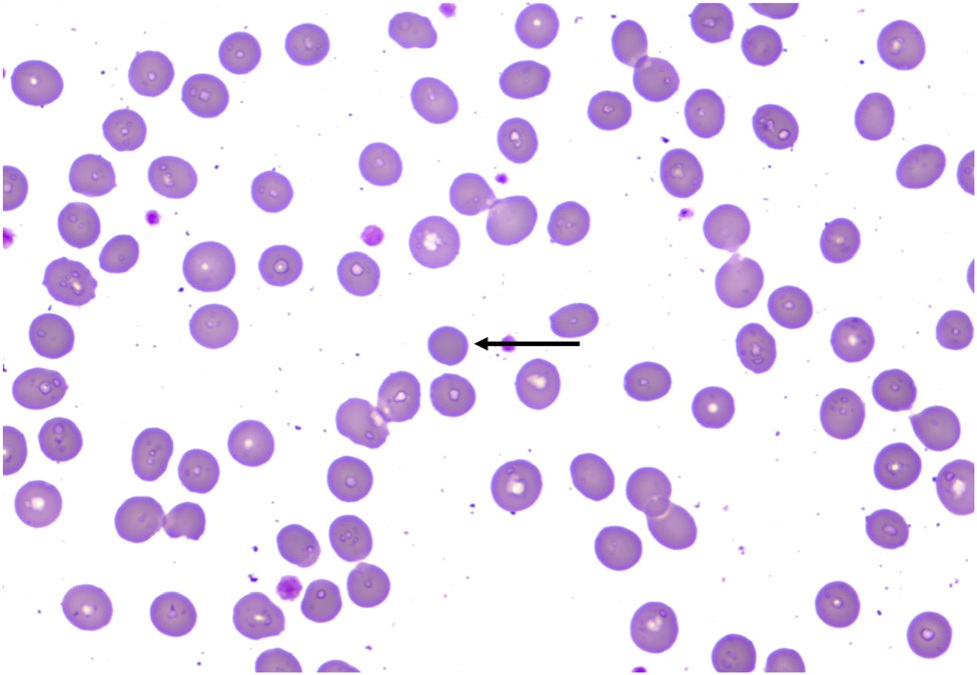
Peripheral blood smear (×1,000): Spherocytes (indicated by arrows).

**Figure 2:**
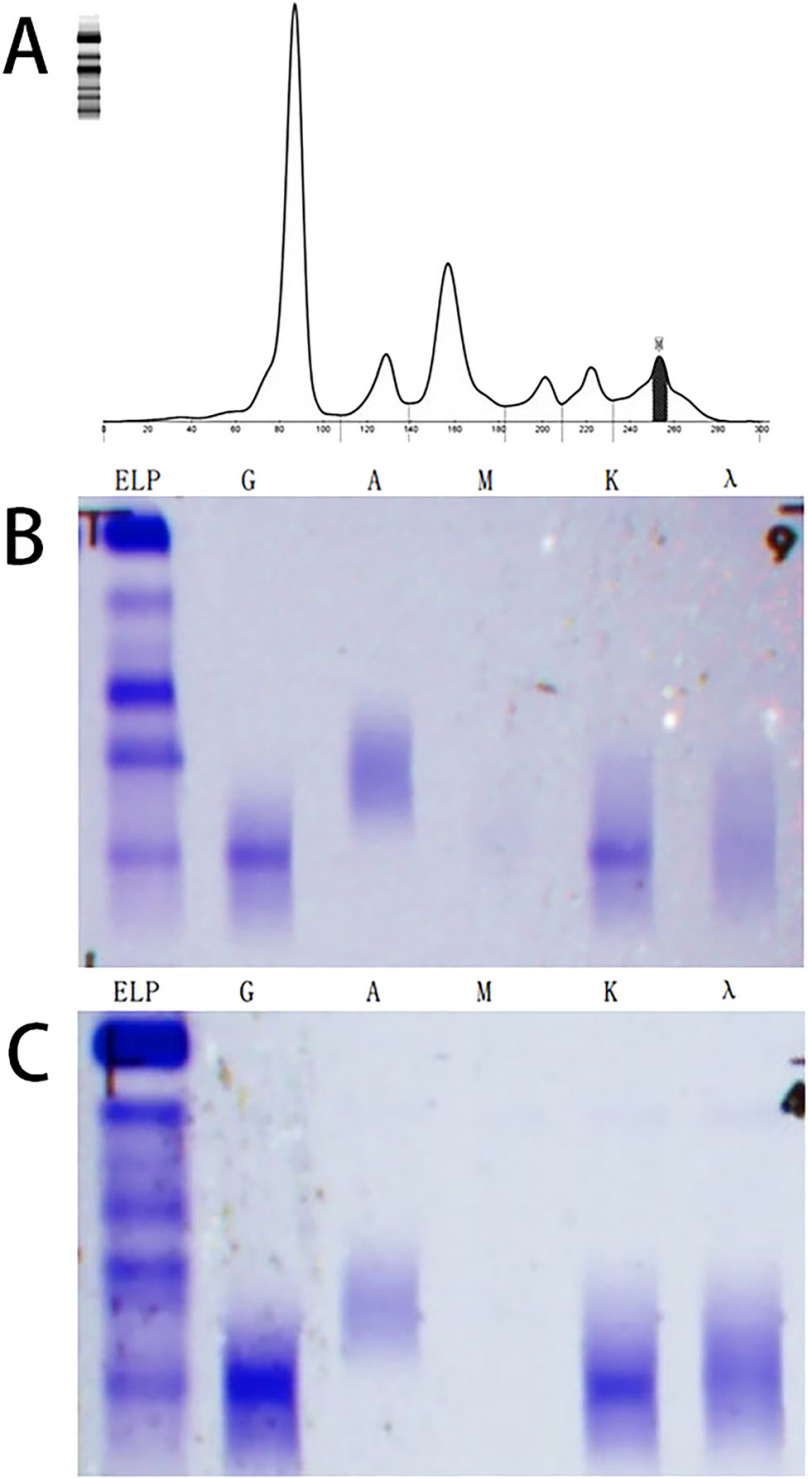
Detection of monoclonal protein in serum and urine. (A) The serum protein electrophoresis revealed an M protein peak in the *γ* region; (B) serum immunofixation identified an IgG-κ monoclonal protein; (C) urine immunofixation also detected an IgG-κ monoclonal protein.

**Figure 3:**
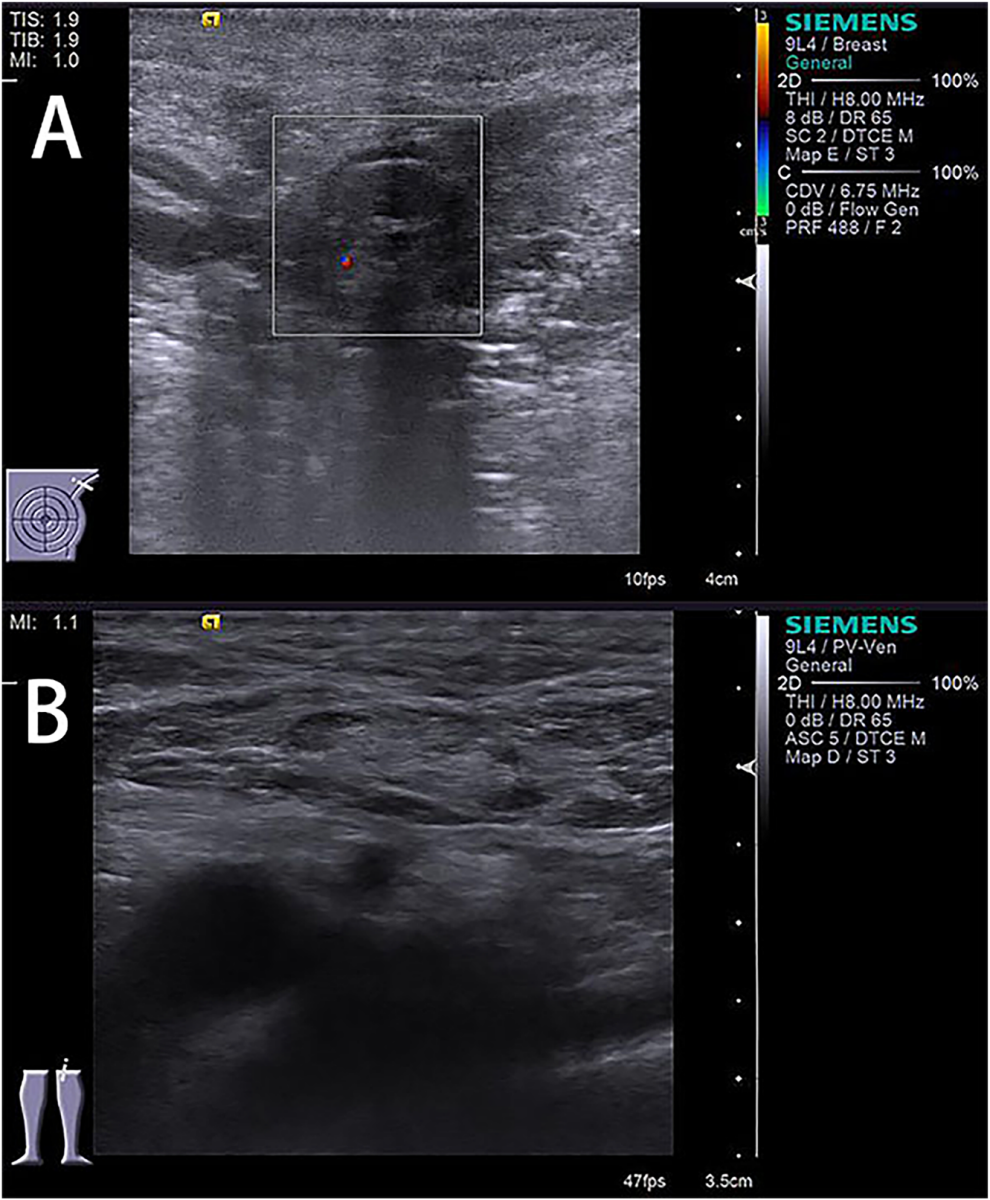
Color Doppler ultrasound images of superficial lymph nodes. (A) Shows enlarged bilateral axillary lymph nodes; (B) displays enlarged bilateral inguinal lymph nodes.

**Figure 4:**
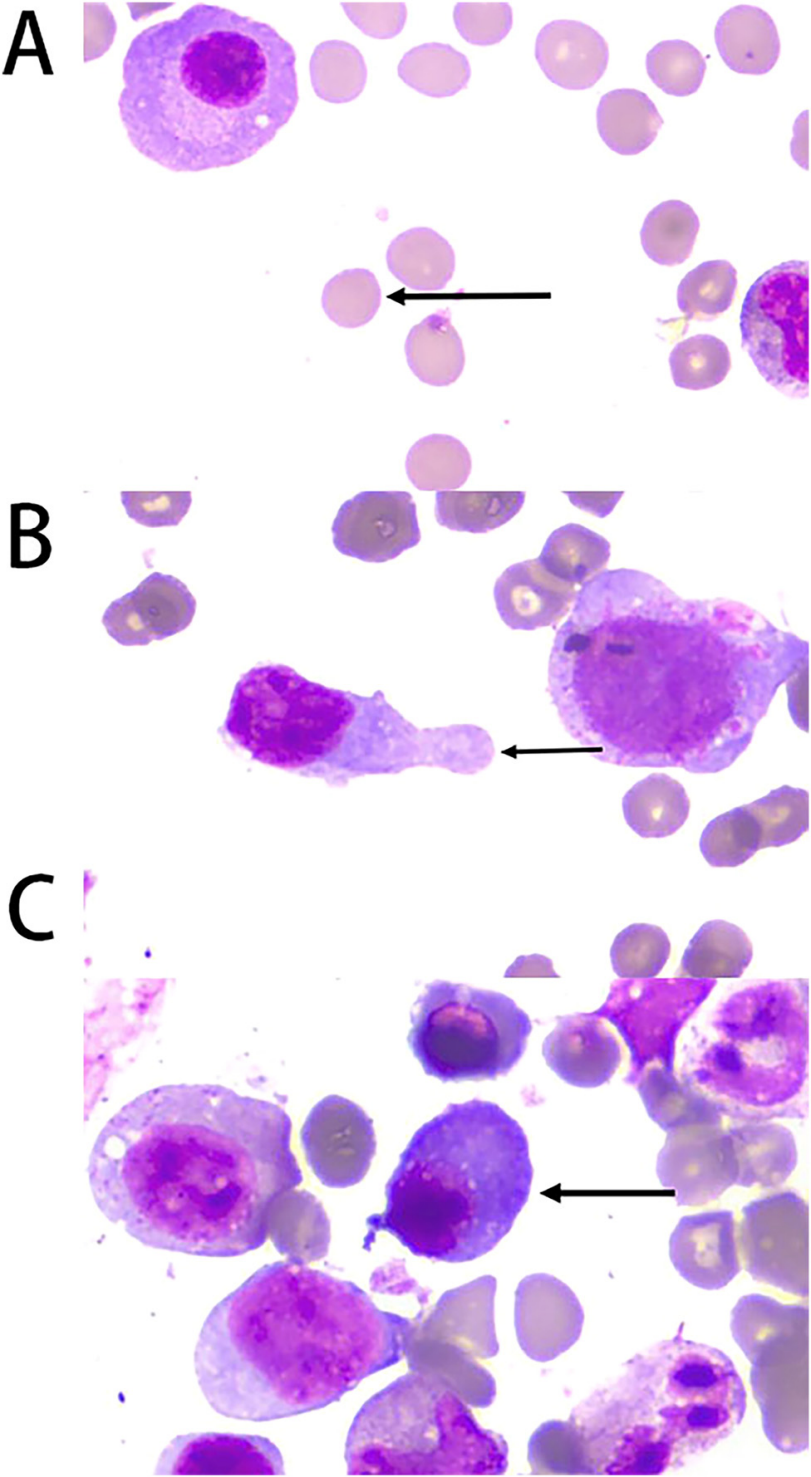
Morphological examination of bone marrow cells (×1,000 magnification). (A) Spherocytes (indicated by arrows); (B) lymphoplasmacytoid cells (indicated by arrows); (C) typical plasma cells (indicated by arrows).

**Figure 5:**
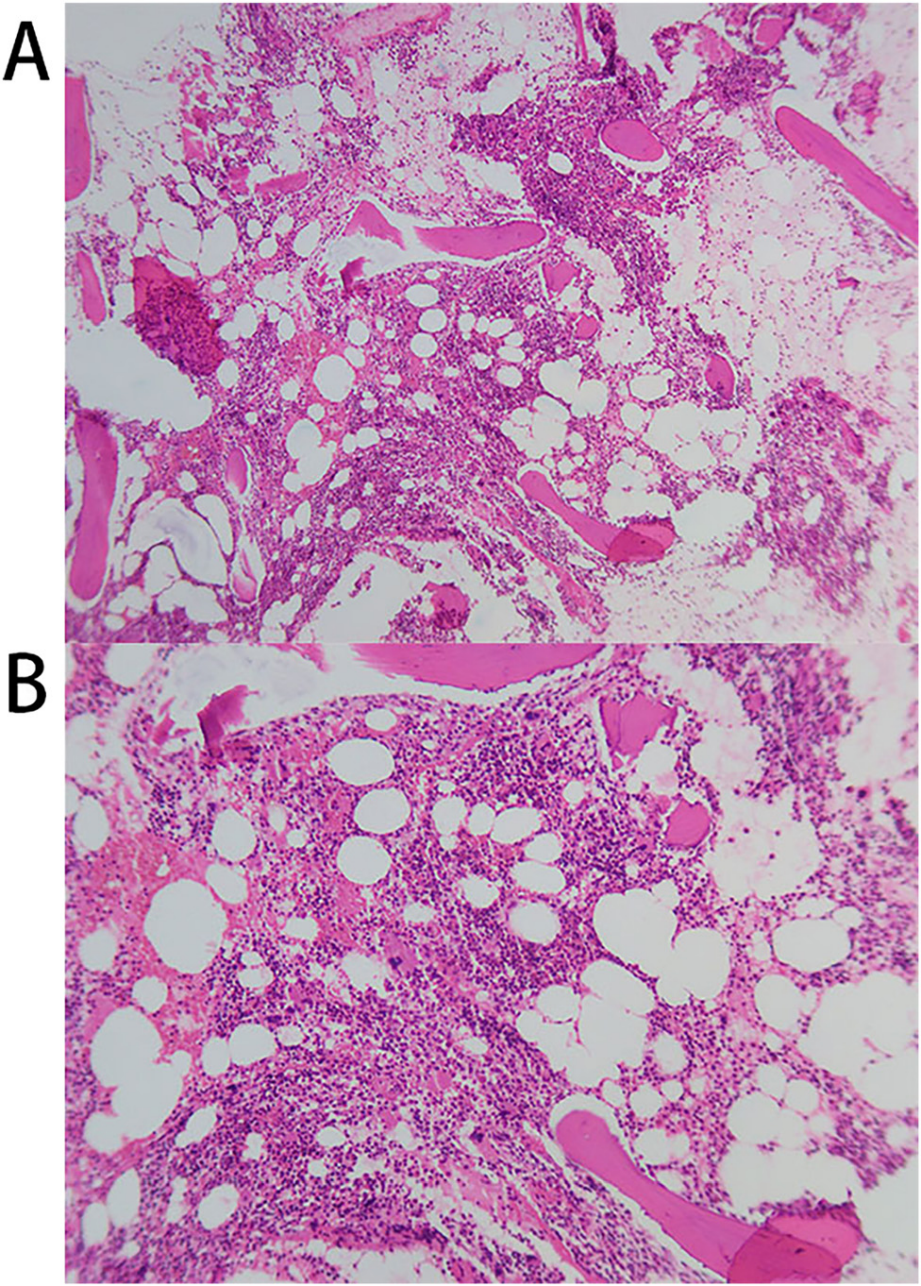
Bone marrow biopsy images of the posterior superior iliac spine. (A) Magnification ×100; (B) magnification ×200.

**Figure 6:**
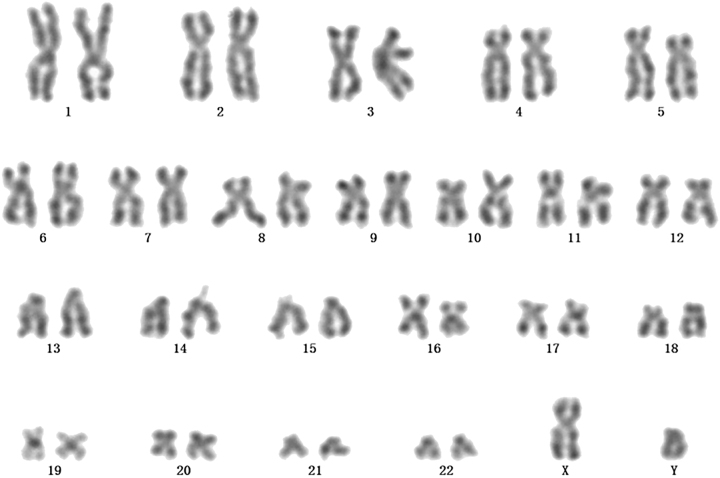
Chromosome analysis diagram of this patient.

A 74-year-old male was admitted to the hospital due to “progressive fatigue for more than two months.” Initially, he experienced unexplained fatigue, and a week before admission, he developed sudden dizziness and speech impairment. He was diagnosed with “cerebral infarction” at another hospital and received thrombolysis and symptomatic treatment. During his stay, continuous blood tests revealed a progressive decline in hemoglobin levels (from 102 to 76 g/L), prompting his transfer to Shengli Oilfield Central Hospital for further evaluation and treatment. The patient has a longstanding history of type 2 diabetes and hypertension, with average control. Laboratory tests indicated moderate anemia: hemoglobin at 80 g/L (130–175 g/L), and white blood cell and platelet counts are normal. The reticulocyte proportion was 2.93 % (0.5–1.5 %), and the reticulocyte production index was 0.752 (>2 indicates sufficient hematopoiesis). Lactate dehydrogenase was 181 U/L (109–245 U/L), total bilirubin was 5.4 μmol/L (0–21 μmol/L), direct bilirubin was 1.6 μmol/L (0–6.8* *μmol/L), and indirect bilirubin was 3.8 μmol/L (0–16 μmol/L). The erythrocyte osmotic fragility test was abnormal (initial hemolysis at 0.52 %, reference range 0.42–0.46 %; complete hemolysis at 0.44 %, reference range 0.32–0.34 %; Supplementary Material 1), and the acidified glycerol dissolution test was 93 s (≥290 s). A peripheral blood smear showed a few spherocytes (Figure 1). Genetic testing for red blood cell mutations revealed a TET24q24 mutation of unknown clinical significance (Supplementary Material 2). Interleukin-6 was 6.23 pg/mL (0–5.3 pg/mL). Ferritin, vitamin B12, and folic acid levels were normal, but renal function was impaired: elevated creatinine with significant proteinuria. Urine microalbumin was 4,465 mg/L (<15 mg/L), accompanied by elevated renal tubular markers (urine α1-microglobulin, urine N-acetyl-*β*-d-glucosaminidase, urine retinol binding protein). Urinary neutrophil gelatinase-associated lipocalin was 13,784 ng/mL (<150 ng/mL). Urine tests showed glucose 2+, occult blood 2+, and protein 3+. Serum protein electrophoresis showed a small M-spike coexisting with polyclonal Ig in *γ* zone (Figure 2A), which was confirmed as IgG-κ paraprotein on immunofixation (Figure 2B). In addition, urine immunofixation revealed κ-type Bence Jones protein (Figure 2C). M protein in peripheral blood was 1.76 g/L (≤5 g/L), and in urine was 408.35 mg/24 h (<150 mg/24 h). Imaging revealed bilateral axillary lymph node enlargement (maximum diameter 1.7 cm) and mild inguinal lymph node enlargement (1.1 cm) (Figure 3), while computed tomography (CT) showed no liver or spleen enlargement. The bone marrow smear reveals hyperactivity. Mature red blood cells vary significantly in size, with spherical forms present. Occasionally, abnormal lymphocytes appear, and plasma cells are scattered (Figure 4). The bone marrow biopsy shows a small infiltration of atypical lymphocytes between the trabeculae, displaying focal and patchy distribution in certain areas, leading to significant compression (Figure 5). Some lymphocytes showed plasmacytoid differentiation. Flow cytometry detected 5.5 % monoclonal small B lymphocytes (CD19+CD20+CD22+CD5−CD10−CD38−CD103−CD123−; L light chain restriction +) and 0.13 % aberrant plasma cells (CD28+CD38+CD138+CD200+CD19−CD20−CD269−; *κ* light chain restriction +) (Supplementary Material 3). Karyotype analysis using cell culture and G-banding methods was normal (46, XY [20]; Figure 6), and the MYD88 L265P mutation was negative. The final diagnosis was lymphoplasmacytic lymphoma (IgG-κ type, MYD88 L265P negative). Despite the abnormal spherocytic morphology, red blood cell osmotic fragility test, and positive acidified glycerol hemolysis test, no pathogenic variations were found in genetic screening for hereditary red blood cell diseases, and the patient showed no hemolytic manifestations. This does not support a diagnosis of hereditary spherocytosis (HS), so LPL with acquired spherocytosis is considered.

The treatment adhered to the “Chinese Guidelines for the Diagnosis and Treatment of Lymphoplasmacytic Lymphoma/Waldenstrom Macroglobulinemia (2022 Edition)” [4]. The patient exhibited fatigue, weight loss, and disease-related cytopenia, meeting the criteria for treatment. A regimen based on a CD20 monoclonal antibody was chosen. Due to the patient’s MYD88 wild-type status and renal insufficiency, bendamustine was excluded. To boost efficacy, zanubrutinib was combined with rituximab, forming the “ZR regimen” (zanubrutinib 160 mg bid D1–28, rituximab 600 mg d1). After six courses of the ZR regimen, the patient’s anemia symptoms improved (hemoglobin 123 g/L), and the enlarged lymph nodes regressed, though renal function continued to decline (Table 1). Currently, rituximab is being used for maintenance therapy, and the patient is under close observation. Given the presence of multiple underlying conditions, long-term follow-up is essential. Notably, despite the absence of hemolytic manifestations, positive serum acidification hemolysis tests and abnormal red blood cell morphology necessitate ongoing monitoring and further evaluation for potential correlation with spherocytosis.

**Table 1:** Temporal changes in laboratory parameters and treatment history in a patient.

	Normal values	2025-02-22	2025-03-27	2025-04-18	2025-05-09	2025-05-28	2025-06-30	2025-09-25	2025-11-01
Peripheral blood									
WBC (×10^9^/L)	3.5–9.5	8.7	3.87	4.21	5.49	2.00	2.55	3.46	3.78
Hemoglobin (g/L)	130–175	80	72	99	109	123	100	123	110
Platelet (×10^9^/L)	125–350	217	156	148	189	163	137	151	152
Urine									
Protein	Negative	3+	3+	3+	3+	3+			3+
Occult blood	Negative	2+	1+	2+	2+	1+			1+
Blood chemistry									
Ca	2.11–2.52	1.95							1.71
Creatinine (mg/dL)	53–123	208.9	228.6	240.7	282.2	277.8	323.9	413.7	515.3
IgG (g/L)	7.00–16.00	6.49				7.27			9.07
IgA (g/L)	0.70–4.00	2.52				2.35			2.72
IgM (g/L)	0.40–2.30	0.25				0.18			0.22
Serum M protein (%)	0.00	4.10	5.40	5.10	5.90	5.50	7.80	9.00	
FLC-κ (mg/L)	6.70–22.40	84.20	90.90	69.60	86.80	86.40	106.00	127.00	
FLC-λ (mg/L)	8.30–27.00	127.00	133.00	111.00	129.00	127.00	171.00	204.00	
κ/λ	0.3100–1.5600	0.6630	0.6835	0.6270	0.6729	0.6803	0.62	0.62	
Treatment		The first course of ZR treatment	The second course of ZR treatment	The third course of ZR treatment	The fourth course of ZR treatment	The fifth course of ZR treatment	The sixth course of ZR treatment	Rituximab monotherapy	Rituximab monotherapy

**Informed consent**: Informed consent has been obtained from all individuals included in this study.

**Ethical approval**: The research related to human use has been complied with all the relevant national regulations, institutional policies and in accordance with the tenets of the Helsinki Declaration, and has been approved by the authors’ Institutional Review Board or equivalent committee.

## 3 Discussion

LPL is a low-grade malignant B-cell lymphoma. Diagnosing LPL requires a thorough evaluation of clinical symptoms, laboratory tests, and pathological features. According to both domestic and international guidelines, the standard diagnostic process for LPL involves a detailed medical history and physical examination, focusing on hepatosplenomegaly and lymph node enlargement. It also includes serum and urine immunofixation electrophoresis for M protein detection, bone marrow puncture and biopsy for morphological and immunophenotypic analysis, and flow cytometry for detecting B-cell and plasma cell clonality. Additionally, cytogenetic and molecular testing, such as MYD88 L265P mutation analysis, is essential. Other B-cell lymphomas and plasma cell diseases must be excluded [1], 5], 6]. In this patient’s case, the diagnostic process adhered strictly to these guidelines. The absence of bone destruction lesions associated with plasma cell proliferation, hypercalcemia, and a positive response to the ZR treatment regimen ruled out multiple myeloma. The tumor cells’ negative expression of CD5 and CD23 excluded small lymphocytic lymphoma. Moreover, the primary infiltration of lymphoma cells in the bone marrow, with a morphology comprising small lymphocytes, plasmacytoid lymphocytes, and plasma cells, without significant involvement of mucosa-associated lymphoid tissue or the spleen, was insufficient for diagnosing marginal zone lymphoma. Serum and urine immunofixation electrophoresis detected IgG-κ M protein, and bone marrow biopsy revealed atypical lymphocytes with plasma cell-like differentiation. Flow cytometry confirmed the presence of monoclonal B cells and plasma cells. Combined with the negative MYD88 L265P mutation and normal karyotype, the final diagnosis was LPL. This comprehensive process effectively ruled out WM and other lymphoma types, underscoring the importance of standardized diagnosis in accurately classifying LPL and guiding treatment.

The MYD88 wild-type, non-IgM type LPL discussed here represents a distinct clinicopathological subtype. Typical WM is marked by the secretion of monoclonal IgM, with clinical symptoms primarily driven by high IgM concentrations. This often results in complications like hyperviscosity syndrome and cryoglobulinemia. However, in this case, the IgG-type LPL, due to its different M protein components, rarely causes hyperviscosity syndrome. Instead, its symptoms are more indicative of blood cell reduction from tumor bone marrow infiltration or organ damage mediated by specific immunoglobulin types, such as the significant kidney damage observed here, differing from typical WM syndromes. This condition overlaps with the clinical manifestations of plasma cell diseases [7]. This fundamental difference necessitates a shift in diagnostic approach: while the presence of the MYD88 L265P mutation clarifies a typical WM diagnosis, diagnosing IgG-type LPL is more complex and requires a careful exclusion of other small B-cell lymphomas and IgG-type multiple myeloma [8]. At the molecular level, the MYD88 L265P mutation is present in over 90 % of WM cases and serves as a crucial molecular marker and therapeutic target [9], 10]. The wild-type status of MYD88 in this instance confirms that non-IgM LPL is a distinct molecular subtype, suggesting its pathogenesis may rely on alternative signaling pathways independent of MYD88. This provides a molecular basis for understanding its potentially unique therapeutic responses.

The primary challenge in this case is to elucidate the causal relationship between LPL and spherocytosis. Through thorough differential diagnosis, we eliminated other common causes: the direct antiglobulin test (Coombs test) was negative, and there was no biochemical evidence of hemolysis, as indicated by normal bilirubin and lactate dehydrogenase levels, making autoimmune hemolytic anemia (AIHA) highly unlikely. Additionally, the patient has no personal or family history of hemolysis, and genetic tests for HS revealed no pathogenic mutations, ruling out an HS diagnosis [2]. Consequently, we strongly suspect that spherocytosis is a paraneoplastic phenomenon induced by LPL. Two potential mechanisms may explain this: First, disruption of the bone marrow microenvironment. Normally, the bone marrow microenvironment supports red blood cell development through the interaction of cell composition, extracellular matrix (ECM), and cytokine networks [11]. In this case, a bone marrow biopsy showed scattered infiltration of atypical lymphocytes between trabeculae, forming focal to fused clusters that significantly compressed surrounding hematopoietic tissue, suggesting disruption of the bone marrow microenvironment. We hypothesize that the abnormal red blood cell morphology is closely linked to bone marrow infiltration. Second, the role of inflammatory factors is significant. Studies indicate that the inflammatory microenvironment associated with lymphoma may induce oxidative stress by releasing cytokines like TNF-α and IL-6, which attack phospholipids and cytoskeletal proteins of red blood cell membranes. This results in decreased membrane flexibility and increased fragility, ultimately forming spherocytes. These abnormal cytokines may also cause ineffective hematopoiesis, inhibit erythroid precursor cell development, and produce morphologically abnormal red blood cells [12]. In this case, the patient’s IL-6 level was slightly elevated, indicating a chronic inflammatory state related to lymphoma, which may lead to spherocytogenesis through the aforementioned mechanism.

The patient was treated with a combination of ZR regimen. This choice was informed by several factors: MYD88 wild-type LPL often shows poor response to standard chemotherapy like bendamustine, whereas Bruton’s tyrosine kinase inhibitors have demonstrated efficacy in MYD88 wild-type WM patients, achieving a total response rate of 88 % [13]. Additionally, rituximab effectively targets and eliminates CD20-positive B cells, which not only reduces tumor burden but may also help alleviate cytopenia [14]. Given the patient’s advanced age and underlying conditions, including diabetes, hypertension, and renal insufficiency, high-intensity chemotherapy was deemed unsuitable. After six courses of the ZR regimen, the patient’s anemia improved, and the enlarged lymph nodes regressed, indicating the regimen’s effectiveness. However, the long-term impact of concomitant spherocytosis, particularly the risk of hemolysis, requires ongoing monitoring. Furthermore, the patient’s renal function continued to decline post-treatment, likely due to underlying diabetic nephropathy. Currently, the endocrinology and nephrology departments are collaboratively managing the patient’s care.

This study has certain limitations. Firstly, as a single case report, its findings cannot be easily generalized to a broader population of lymphoplasmacytic lymphoma. Secondly, while abnormalities in the red blood cell membrane were observed, the limitations of detection conditions prevented an in-depth analysis of the protein composition of the red blood cell membrane, the cytoskeleton structure, or oxidative stress markers. Consequently, the specific molecular mechanism behind spherocytic formation remains unclear. Given these limitations, future research should explore multiple directions: multi-center case summary analyses could systematically compare clinical characteristics, molecular phenotypes, and treatment responses among LPL patients with or without spherocythemia, thereby establishing a more valuable clinical prediction model for differentiation. Additionally, applying proteomics and single-cell sequencing techniques could analyze protein expression profiles of erythrocyte membranes and the characteristics of the bone marrow hematopoietic microenvironment in LPL patients, aiming to identify specific biomarkers related to abnormal erythrocyte morphology. Based on these findings, intervention strategies for tumor-related red blood cell membrane damage could be explored, such as evaluating the potential application of antioxidants or cytokine inhibitors to improve red blood cell quality.

In summary, this case highlights a complex diagnostic and therapeutic scenario involving the coexistence of hematological malignancies (LPL) and acquired erythrocyte morphological abnormalities (spherocytosis). The diagnostic process combines the standard LPL diagnostic procedure with the differential diagnosis of acquired erythrocyte membrane defects, underscoring the importance of multidisciplinary collaboration in tackling complex hematological challenges. The presence of multiple chronic diseases in patients further complicates treatment, necessitating that clinicians carefully balance efficacy with the management of comorbidities when developing anti-tumor strategies and implementing truly individualized treatment plans.

## 4 Conclusions

This case report details an elderly patient with LPL and spherocytosis, where the red blood cell abnormalities are likely due to tumor-related membrane defects rather than typical HS or AIHA. The ZR combined protocol has shown excellent disease control. This case underscores the importance of comprehensive diagnostic assessment and integrated treatment for patients with complex comorbidities. It also highlights the critical role of multidisciplinary collaboration and long-term follow-up in optimizing clinical outcomes. Future research should focus on accumulating more cases and investigating mechanisms to clarify the association between LPL and spherocytosis and to establish the best treatment strategy.

## Supplementary Material

Supplementary Material

Supplementary Material

Supplementary Material

Supplementary Material
