# Supplementary material for: A case report of lymphoplasmacytic lymphoma with spherocytosis
Source: Open Life Sci. 2026 Mar 2;21(1):20251286. doi: 10.1515/biol-2025-1286 (PMC12952209; doi:10.1515/biol-2025-1286)
Supplement: Supplementary file 1 — Supplementary Material [file j_biol-2025-1286_suppl_001.pdf]

打印时间：2025-04-16 00:50:12
临检实验室
红细胞渗透脆性试验

胜利油田中心医院检验科

质评合格 山东HR
条码编号：[redacted]

姓 名：[redacted]　登记号：[redacted]　申请科室：血液内科一病区　申请医师：[redacted]　样本号：12
性 别：男　住院号：[redacted]　标本类型：全血/静脉血　初步诊断：头晕,脑梗死(急性期),中度贫血,社区获得性肺炎，非重症,2...
年 龄：74岁　床 号：34　申请时间：2025-02-25 08:20　采集时间：2025-02-26 06:07

No	项目名称	缩写	结果	单位		参考范围
1	开始溶血	KSRX	0.52	%	↑	0.42-0.46
2	完全溶血	WQRX	0.44	%	↑	0.32-0.34
3	正常对照开始溶血	DZKSRX	0.46	%		0.42-0.46
4	正常对照完全溶血	DZWQRX	0.34	%		0.32-0.34

报告评价：　　医嘱备注：(工作日采集)

接收时间：2025-02-26 08:56　报告时间：2025-02-26 13:54　检验者：[redacted]　审核者：[redacted]

注：“★”标记项目为京津冀鲁地区检验结果互认项目，“*”标记项目为山东地区检验结果互认项目，“危”代表危急值，如有疑问请7日内与检验科 8770171 联系，地址济南路31号。
