# Supplementary material for: A case report of lymphoplasmacytic lymphoma with spherocytosis
Source: Open Life Sci. 2026 Mar 2;21(1):20251286. doi: 10.1515/biol-2025-1286 (PMC12952209; doi:10.1515/biol-2025-1286)
Supplement: Supplementary file 2 — Supplementary Material [file j_biol-2025-1286_suppl_002.pdf]

红细胞相关疾病基因突变筛查检测报告

标本号：[redacted]　申请序号：[redacted]　住院号：

姓名：[redacted]　性别：男　年龄：74
科室：血液科　院别：胜利油田中心医院
标本类型：外周血　送检医师：[redacted]　采集日期：2025-03-04 12:33
诊断：淋巴浆细胞淋巴瘤 Igg型

一、检测结果：

1. 预测为致病的位点突变检测结果

基因	基因组位置	内含子/外显子	变异信息	dbSNP	杂合性	相关疾病/表型（遗传方式）	ACMG分级
--	--	--	--	--	--	--	--

2. 预测为疑似致病的位点突变检测结果

基因	基因组位置	内含子/外显子	变异信息	dbSNP	杂合性	相关疾病/表型（遗传方式）	ACMG分级
--	--	--	--	--	--	--	--

3. 临床意义不明的位点突变检测结果

基因	基因组位置	内含子/外显子	变异信息	dbSNP	杂合性	相关疾病/表型（遗传方式）	ACMG分级
TET24 q24	chr4:106196297C>A	exon11	NM_001127208 c.4630C>A p.P1544T	--	杂合	免疫缺陷75型(AR)	意义未明

注：此报告仅对该份被检测样本有效，若有疑问，请在收到结果后的7天内与我们联系！
地址：天津市静海区团泊新城西区健康产业园北华北路1号
电话：022-68647136;13682159519

红细胞相关疾病基因突变筛查检测项目

检测内容：红细胞相关疾病涉及的142个基因的全部外显子区域及其他特殊致病区域

基因列表如下：

ABCB6	ABCB7	ABCD4	ABCG5	ABCG8	ADA	AK1
ALAD	ALAS2	ALDOA	AMMECR1	AMN	ANK1	ATP11C
ATP7B	ATRX	BMP2	BPGM	CD59	CDAN1	CDIN1
CLPX	COQ2	COX4I2	CP	CPO	CPOX	CUBN
CYB5A	CYB5R3	DHFR	EGLN1	EPAS1	EPB41	EPB42
EPO	EPOR	FECH	FTH1	FTL	G6PD	GATA1
GCLC	GLRX5	GPI	GPX1	GSR	GSS	HAMP
HBA1	HBA2	HBB	HBG1	HBG2	HFE	HJV
HK1	HMOX1	HP	HSCB	HSPA9	JAK2	KCNN4
KIF23	KLF1	LARS2	LCAT	LMBRD1	LPIN2	MMAB
MMACHC	MMADHC	MTR	MTRR	NT5C3A	PANK2	HMBS
PFKL	PFKM	PGK1	PIEZO1	PIGA	PIGT	PKLR
PNP	PPOX	PUS1	RACGAP1	RHAG	SEC23B	SLC11A2
SLC19A2	SLC25A38	SLC2A1	SLC40A1	SLC46A1	SLC4A1	SPTA1
SPTB	STEAP3	TCN2	TF	TFR2	TMPRSS6	TPI1
TRNT1	UMPS	UROD	UROS	VHL	VPS4A	XK
YARS2	VPS13A	SLCO1B1	SLCO1B3	SH2B3	NDUFB11	SF3B1
CALR	MMAA	PRDX1	SFXN4	MPL	MTHFR	TET2
UGT1A1	TBXAS1	THBD	C3	CFB	CFH	CFHR1
CFHR3	CFI	DGKE	CD46	HBD	HBE1	CD55
ADAMTS13	WAS					

检验者：[redacted] **审核者：**[redacted] **报告日期： 2025-03-12 09:56**

注：此报告仅对该份被检测样本有效，若有疑问，请在收到结果后的7天内与我们联系！

地址：天津市静海区团泊新城西区健康产业园北华北路1号
电话：022-68647136;13682159519
