# Supplementary material for: A case report of lymphoplasmacytic lymphoma with spherocytosis
Source: Open Life Sci. 2026 Mar 2;21(1):20251286. doi: 10.1515/biol-2025-1286 (PMC12952209; doi:10.1515/biol-2025-1286)

金域医学 KingMed Diagnostics

流式细胞免疫荧光分析结果报告单

标本条码		医　院	胜利油田中心医院		
病人姓名		科　室	血一	实验号	
性　别	男	房/床号	34	门诊/住院号	
年　龄	74岁	接收时间	2025-02-22 12:30:49	申请医生	
送检材料	骨髓	采样时间	2025-02-22 10:00:00	医院标识	
标本情况	无肉眼可见异常			联系电话	
临床诊断	贫血待查				

检测项目： 急慢性白血病/NHL/MDS全面免疫分型检测(40CD)

细胞群体	所占比率（%）	细胞系列/表型分析
淋巴细胞 Lymphoid	12.74	可见克隆性B淋巴细胞，其免疫表型详见解释与意见部分。
粒细胞 Grans	75.81	相对比例升高，其免疫表型CD13，CD16，CD15，CD11b未见明显表达紊乱，可见少量浆细胞。
单核细胞 Monos	3.96	相对比例正常。
CD45弱表达细胞	0.54	未见明显异常。
CD45阴性表达细胞	6.95	主要为有核红细胞和细胞碎片。

样本活性(Sample Viability)： 99 %

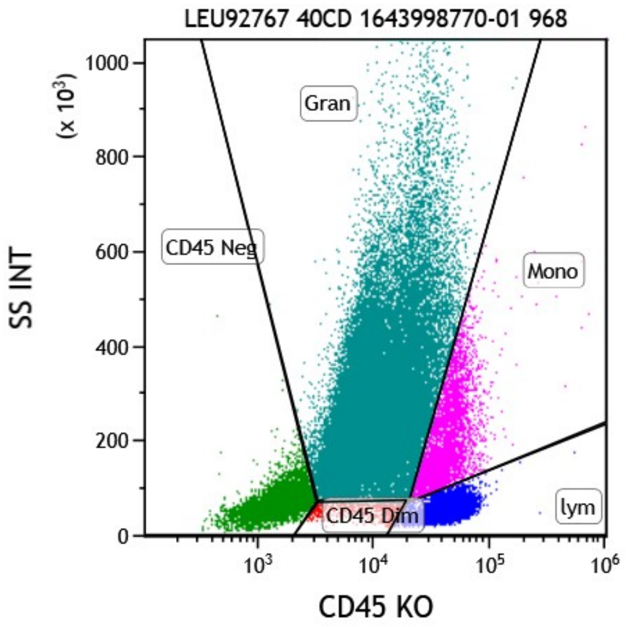

分析结论(Conclusion)：

送检标本流式分析可见5.50%CD5阴性CD10阴性单克隆小B淋巴细胞（L+）及0.13%的单克隆浆细胞（K+）；请结合骨髓活检、免疫固定电泳和基因等结果综合考虑。

解释与意见(Interpretation & Comments)：

送检标本流式分析可见5.50%异常B淋巴细胞，其FSC较小，其免疫表型为CD19+，CD20+，CD22+，CD5-，CD10-，CD11c-，CD25-，CD38-，CD103-，CD123-，胞内免疫球蛋白Lambda轻链限制性表达，提示为单克隆B细胞；T细胞占淋巴细胞53.60%，CD4:CD8=0.56，未见明显异常；NK细胞占淋巴细胞4.85%，未见明显异常；可见异常浆细胞占有核细胞总数0.13%，其免疫表型为CD28+，CD38+，CD200+，CD138+部分，CD19-，CD20-，CD269-，胞内免疫球蛋白Kappa轻链限制性表达，提示为单克隆浆细胞；另可见0.11%浆细胞，胞内免疫球蛋白Kappa/Lambda轻链呈多克隆表达，提示为正常浆细胞；CD34+细胞占有核细胞总数的0.46%，其免疫表型未见明显异常；粒细胞相对比例升高，其免疫表型CD13，CD15，CD16，CD11b未见明显表达紊乱。

此报告检测的CD(Markers Run)：

CD2、CD3、CD4、CD5、CD7、CD8、CD10、CD11b、CD11c、CD13、CD14、CD15、CD16、CD19、CD20、CD22、CD25、CD28、CD33、CD34、CD36、CD38、CD41、CD203c、CD56、Cytokeratin、CD64、CD71、CD117、CD123、CD138、CD200、CD269、HLA-DR、7AAD、CD103、CD123、CD45、Kappa、Lambda等共40次。

本报告为非诊断性报告，本报告所有检测结果和信息仅供临床医生参考使用。

流式细胞免疫荧光分析结果报告单

标本条码		医　院	胜利油田中心医院		
病人姓名		科　室	血一	实验号	
性　别	男	房/床号	34	门诊/住院号	
年　龄	74岁	接收时间	2025-02-22 12:30:49	申请医生	
送检材料	骨髓	采样时间	2025-02-22 10:00:00	医院标识	
标本情况	无肉眼可见异常			联系电话	
临床诊断	贫血待查				

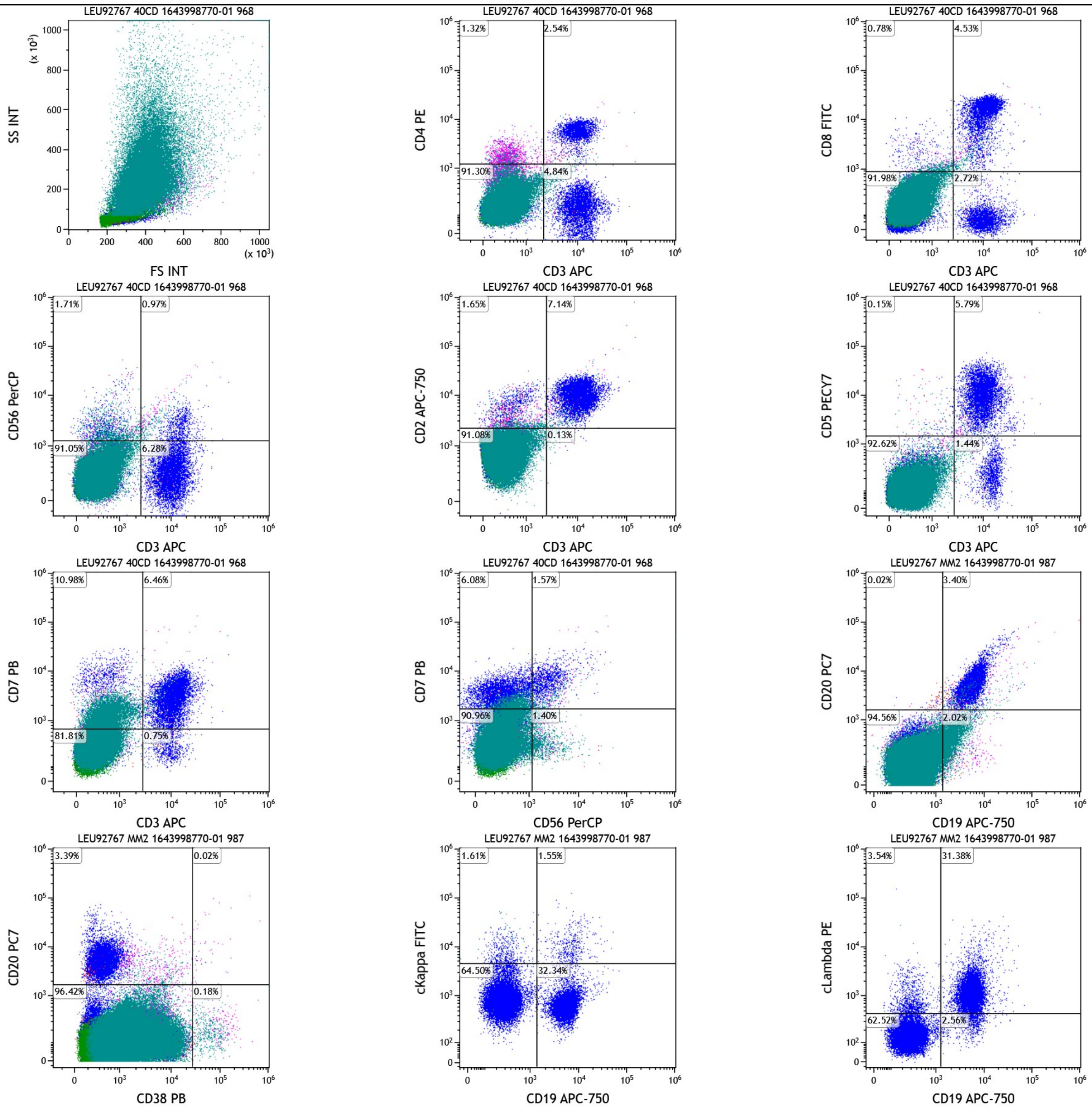

本报告为非诊断性报告，本报告所有检测结果和信息仅供临床医生参考使用。

本检测仅对来样负责，如果对结果有疑义，请在报告发布后7天内与我们联系，多谢合作！

收样点：胜利油田中心医院-检验科

主检：

主检实验室：济南金域

报告日期：2025-02-24 14:38:37

网址： www. ingme .com.cn　电话：4001-111-120
地址：　山东省济南市高新区开拓路2333号

流式细胞免疫荧光分析结果报告单

标本条码		医　院	胜利油田中心医院		
病人姓名		科　室	血一	实验号	
性　别	男	房/床号	34	门诊/住院号	
年　龄	74岁	接收时间	2025-02-22 12:30:49	申请医生	
送检材料	骨髓	采样时间	2025-02-22 10:00:00	医院标识	
标本情况	无肉眼可见异常			联系电话	
临床诊断	贫血待查				

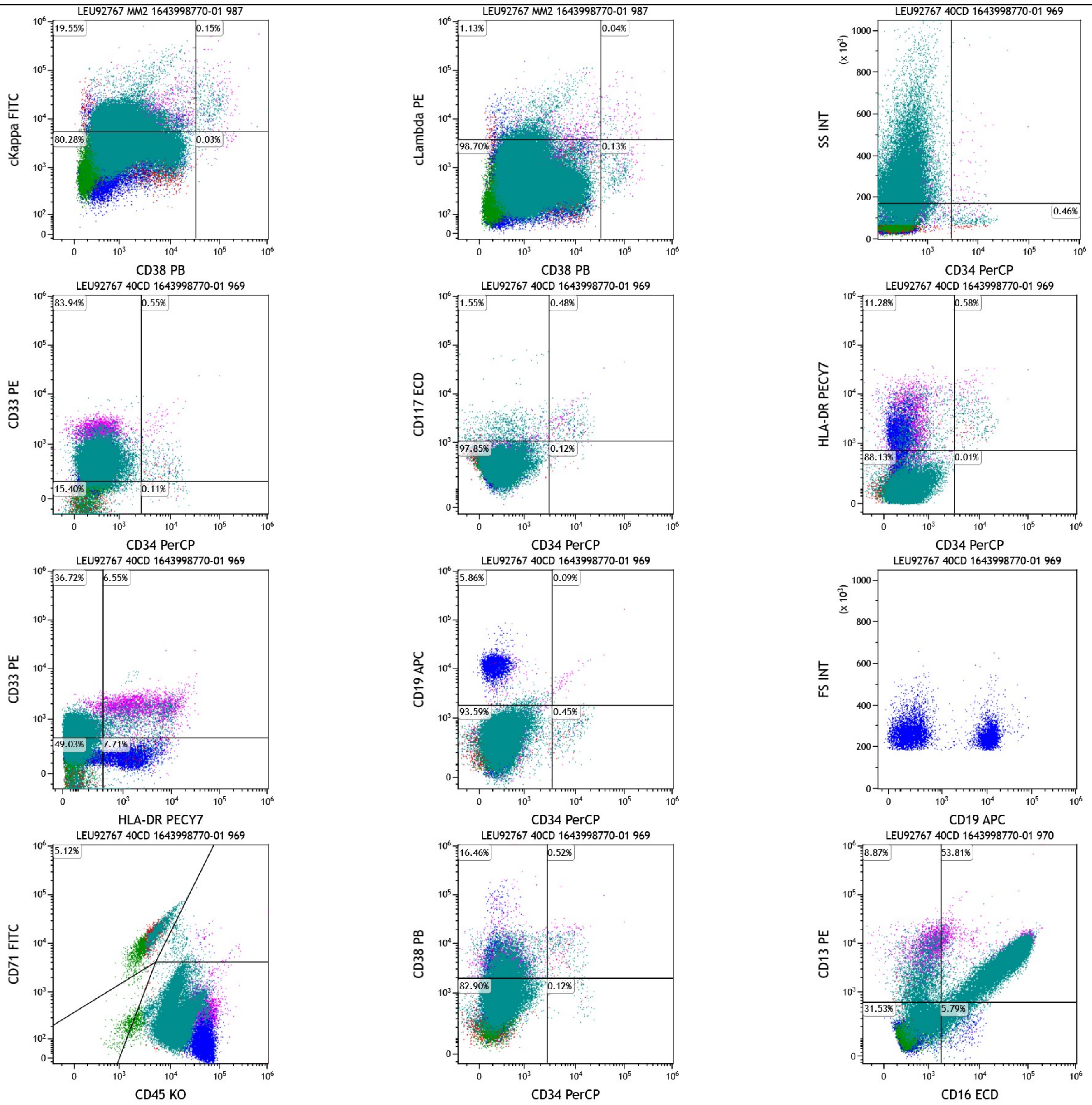

本报告为非诊断性报告，本报告所有检测结果和信息仅供临床医生参考使用。

流式细胞免疫荧光分析结果报告单

标本条码		医　院	胜利油田中心医院		
病人姓名		科　室	血一	实验号	
性　别	男	房/床号	34	门诊/住院号	
年　龄	74岁	接收时间	2025-02-22 12:30:49	申请医生	
送检材料	骨髓	采样时间	2025-02-22 10:00:00	医院标识	
标本情况	无肉眼可见异常			联系电话	
临床诊断	贫血待查				

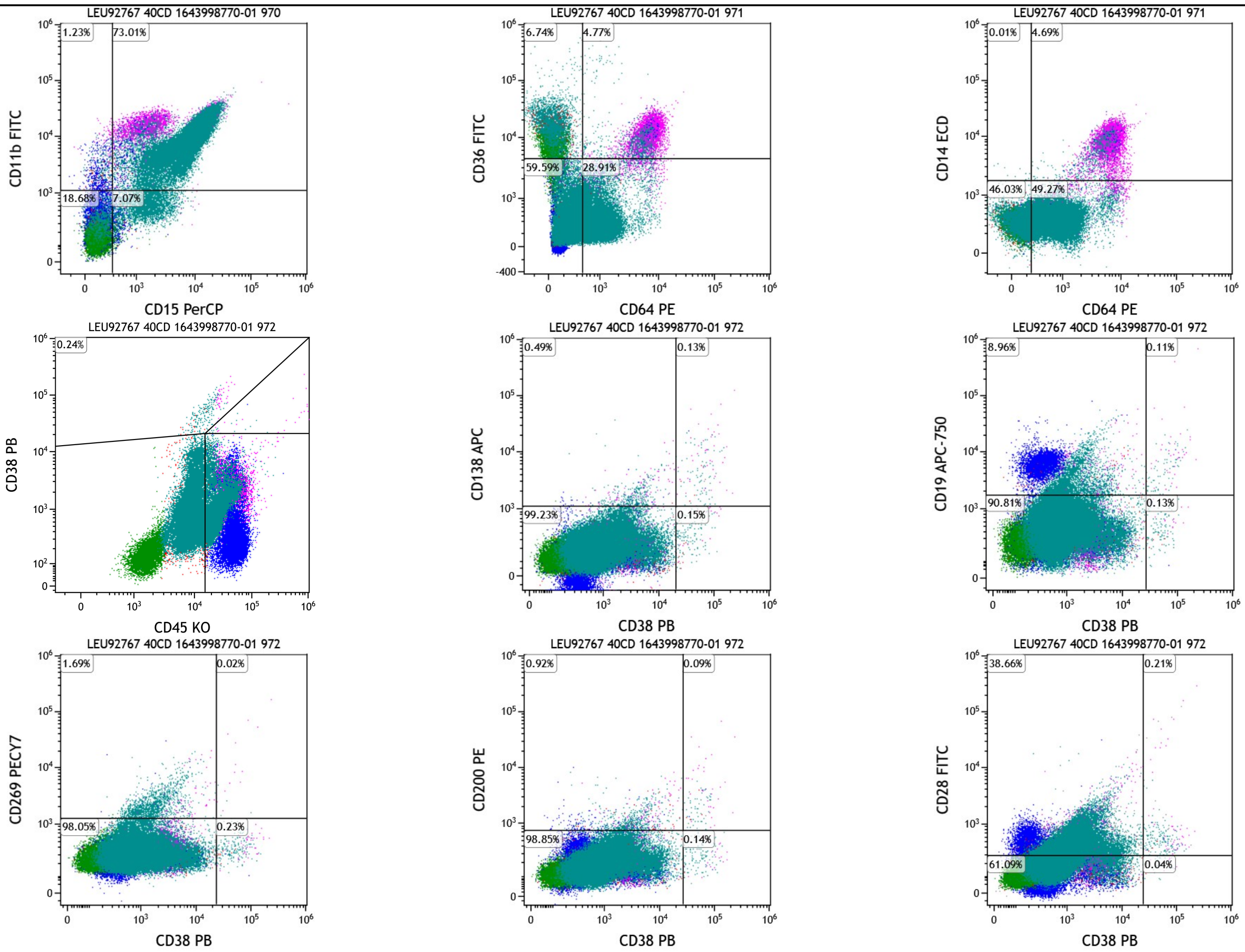

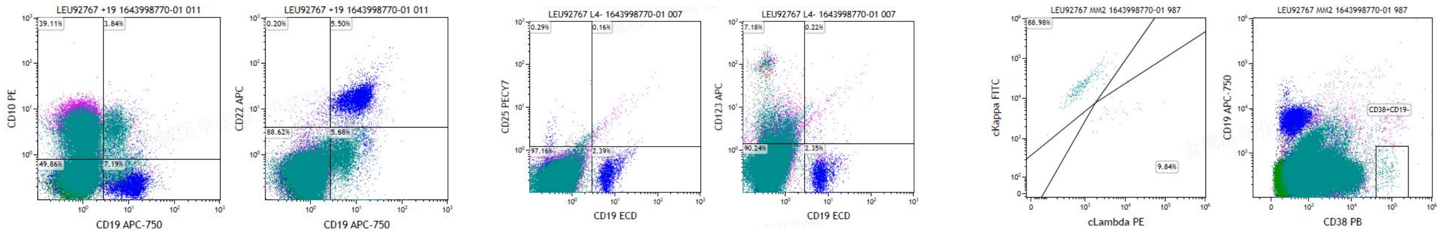

本报告为非诊断性报告，本报告所有检测结果和信息仅供临床医生参考使用。

济南金域医学检验中心
JINAN KINGMED CENTER FOR CLINICAL LABORATORY

流式细胞免疫荧光分析结果报告单

标本条码		医　院	胜利油田中心医院		
病人姓名		科　室	血一	实验号	
性　别	男	房/床号	34	门诊/住院号	
年　龄	74岁	接收时间	2025-02-22 12:30:49	申请医生	
送检材料	骨髓	采样时间	2025-02-22 10:00:00	医院标识	
标本情况	无肉眼可见异常			联系电话	
临床诊断	贫血待查				

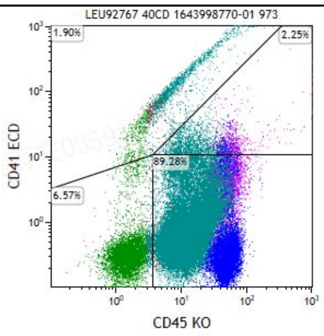

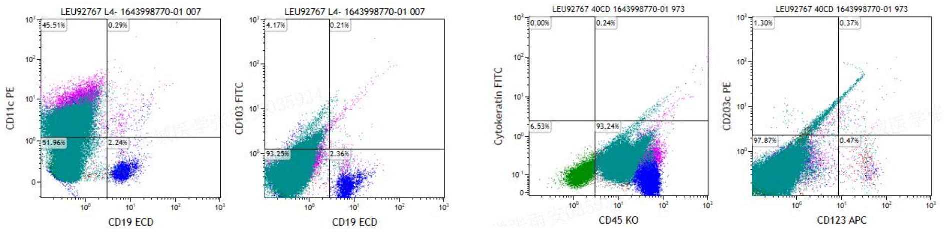

Supplement: Supplementary file 3 — Supplementary Material [file j_biol-2025-1286_suppl_003.pdf]
