# Supplementary material for: A case report of lymphoplasmacytic lymphoma with spherocytosis
Source: Open Life Sci. 2026 Mar 2;21(1):20251286. doi: 10.1515/biol-2025-1286 (PMC12952209; doi:10.1515/biol-2025-1286)
Supplement: Supplementary file 4 — Supplementary Material [file j_biol-2025-1286_suppl_004.docx]

**Supplementary material 1**. Report of erythrocyte osmotic fragility test.

**Supplementary material 2.** Gene mutation detection report for red blood cell-related diseases.

**Supplementary material 3.** Report of flow cytometric immunofluorescence analysis results.
